# Characterization of Insect Resistance Loci in the USDA Soybean Germplasm Collection Using Genome-Wide Association Studies

**DOI:** 10.3389/fpls.2017.00670

**Published:** 2017-05-15

**Authors:** Hao-Xun Chang, Glen L. Hartman

**Affiliations:** ^1^Department of Plant, Soil, and Microbial Sciences, Michigan State UniversityEast Lansing, MI, USA; ^2^United States Department of Agriculture - Agricultural Research Service, University of IllinoisUrbana, IL, USA

**Keywords:** genome-wide association study, soybean insect resistance

## Abstract

Management of insects that cause economic damage to yields of soybean mainly rely on insecticide applications. Sources of resistance in soybean plant introductions (PIs) to different insect pests have been reported, and some of these sources, like for the soybean aphid (SBA), have been used to develop resistant soybean cultivars. With the availability of SoySNP50K and the statistical power of genome-wide association studies, we integrated phenotypic data for beet armyworm, Mexican bean beetle (MBB), potato leafhopper (PLH), SBA, soybean looper (SBL), velvetbean caterpillar (VBC), and chewing damage caused by unspecified insects for a comprehensive understanding of insect resistance in the United States Department of Agriculture Soybean Germplasm Collection. We identified significant single nucleotide (SNP) polymorphic markers for MBB, PLH, SBL, and VBC, and we highlighted several leucine-rich repeat-containing genes and myeloblastosis transcription factors within the high linkage disequilibrium region surrounding significant SNP markers. Specifically for soybean resistance to PLH, we found the PLH locus is close but distinct to a locus for soybean pubescence density on chromosome 12. The results provide genetic support that pubescence density may not directly link to PLH resistance. This study offers a novel insight of soybean resistance to four insect pests and reviews resistance mapping studies for major soybean insects.

## Introduction

Insect damage is one of the major limiting factors for soybean (*Glycine max* (L.) Merr.) production as insects vector viruses and cause damage by feeding on foliage, vascular sap, stems, roots, pods, and seeds (Steffey, 2015). In order to manage yield losses, application of insecticides has often been the first tool in management. For example, insecticide usage increased in response to soybean aphid (SBA, *Aphis glycines* Matsumura) dissemination in the north central region of the USA (Coupe and Capel, 2015), where 80% of soybean production occurs. Insect damage also devastated production in the southern parts of the USA where 234 million dollars of soybean losses were reported despite investing 279 million dollars on insect management (Musser et al., 2016; Ortega et al., 2016). Soybean is one of the most important field crops for providing dietary protein and oil worldwide, and it is estimated that a 50% increase in soybean production is needed to meet the population expansion by 2030 (Hartman et al., 2011; Ainsworth et al., 2012). In addition to insecticidal use, an alternative way to control insect damage is through genetic resistance. Insect resistance has been widely studied and applied in breeding program for several crops (Edwards and Singh, 2006; de Morals and Pinheiro, 2012). Characterization of novel resistance to different insects may be essential to sustain soybean productivity.

Plants defend themselves against insects in a number of ways and mechanisms of resistance have been described as antibiosis, non-preference, and tolerance or the interaction of these three factors (Painter, 1941). While antibiosis describes the ability of resistant plants to restrict insect growth or propagation, non-preference or later referred to as antixenosis, deters insects from feeding and propagating on resistant plants (Parrott et al., 2008). Both antibiosis and antixenosis resistance could be genetically governed by the same locus in a resistant genotype. For example, one of the major insect resistance quantitative trait loci (QTL) in soybean is the QTL-E (SoyBase QTL name: corn earworm 8-1) on chromosome (chr) 15, on linkage group E (LG-E) (Terry et al., 2000; Hulburt et al., 2004). QTL-E contributes 26% of the antibiotic effect and 20% of the antixenotic effect of resistance to corn earworm (CEW, *Helicoverpa zea* Boddie) (Boerma and Walker, 2005). QTL-E has been mapped to the same location to the *Pb* locus that determines the tip phenotype of pubescence, sharp (*Pb*) or blunt (*pb*) (Palmer and Xu, 2008; Parrott et al., 2008; Ortega et al., 2016). It has been shown that the *Pb* locus provides antixenotic resistance by discouraging insect feeding on soybeans with sharp-tipped pubescence (Hulburt et al., 2004), a trait which is rare in domesticated soybean but common in wild soybean (*G*. *soja*) (Broich and Palmer, 1981). Another insect resistance QTL is QTL-M (SoyBase QTL name: corn earworm 1-1) on chr 7 (LG-M) which accounts for 22% of the antibiotic effect and 37% of the antixenotic effect of resistance to CEW (Rector et al., 1998, 1999, 2000), as well as resistance to other insects including several lepidopteran insects such as soybean looper (SBL, *Pseudoplusia includens* Walker), velvetbean caterpillar (VBC, *Anticarsia gemmatali*s Hübner) and a coleopteran insect, Mexican bean beetle (MBB, *Epilachna varivestis* Mulsant). QTL-M also exhibits synergistic epistasis to QTL-G on chr 18 (LG-G, SoyBase QTL name: corn earworm 6-1) that conditions antibiosis and to QTL-H on chr 12 (LG-H, SoyBase QTL name: corn earworm 1-2) that conditions antixenosis (Rector et al., 1998, 2000; Zhu et al., 2008). Accordingly, QTL-M has become a major breeding target for soybean, and a follow up study that fine-mapped QTL-M between Sat_258 and Satt702 in a 0.25 cM interval (Parrott et al., 2008).

Along with resistance to defoliator insects, resistance to piercing-sucking insects in soybean has also been well documented. Aphid resistance in soybean was found to be antibiotic and antixenotic in varieties “Dowling” and “Jackson,” but only antixenotic in the variety “PI 71506” (Hill et al., 2004). The antibiotic QTL in “Dowling” and “Jackson” were mapped approximately to QTL-M, and were assigned as *Rag1* (Resistance to *Aphis glycines* 1) and *Rag*, respectively (Li et al., 2007). Other SBA resistance QTL include antibiotic *Rag2* on chr 13 (Mian et al., 2008; Hill et al., 2009), antixenotic *Rag3* and *Rag3b* on chr 16 (Zhang et al., 2010, 2013), and two antibiotic recessive QTL (*rag1c* and *rag4*) on chr 7 and 13, respectively (Zhang et al., 2009). Fine mapping efforts on *Rag1* discovered two nucleotide binding leucine-rich repeat (NBS-LRR) genes among 13 candidate genes in a 115 Kb interval on chr 7, and fine mapping on *Rag2* identified one NBS-LRR gene along with seven candidate genes in a 54 Kb region on chr 13 (Kim et al., 2010a,b). However, instead of the NBS-LRR gene (Glyma13g26000) within the *Rag2* region, another NBS-LRR (Glyma13g25970) that locates on the border of *Rag2* was proposed to be the candidate resistance gene based on differential expression analyses (Brechenmacher et al., 2015).

The resolution of linkage mapping has been limited by the density of traditional DNA markers, such as simple sequence repeat (SSR), and by the limited recombination that occurs during bi-parental crossing. In addition, another disadvantage of linkage mapping is the monotonous resistance source from one of the parents. The majority of resistance to defoliator insects (CEW, MBB, SBL, and VBC) was derived from three Japanese accessions (PI 171451, PI 227687, and PI 229358) (Parrott et al., 2008) and resistances to SBA were reported in a few accessions (Dowling, PI 200538, PI 567324, PI 567537, PI 567541B, PI 567543C, PI 567597C, and PI 587732) (Hill et al., 2004; Bales et al., 2013; Jun et al., 2013; Kim et al., 2014). However, SBA biotypes that overcome different *Rag* resistance were reported in the field (Hill et al., 2010), and thus identification of additional resistance sources is important. With the advance in biotechnology, methods such as Affymetrix GeneChip and genotyping-by-sequencing enabled single nucleotide polymorphisms (SNPs) have become useful as novel genetic markers (Barabaschia et al., 2016). High-density and high-quality SNPs across the entire genome empowers the genome-wide association study (GWAS), which relies on linkage disequilibrium (LD) that has remained through historical recombination in a diverse population. Accordingly, GWAS provides better mapping resolution and also detects multiple genetic sources in a germplasm collection. The power of GWAS has been well recognized in numerous studies, including those on soybean agronomic traits (Zhou et al., 2015) and on soybean disease resistance (Chang et al., 2016a,b,c). There are fewer GWAS focusing on insect resistance in soybean (Wang et al., 2015; Liu et al., 2016).

The goal of this study was to integrate the insect resistance phenotypes in the Germplasm Resources Information Network (GRIN) (www.ars-grin.gov) and the SNPs in the Soybean Germplasm Collection maintained by the United States Department of Agriculture Agricultural Research Service (USDA-ARS). A comprehensive GWAS for six soybean insects, including beet armyworm (BAW, *Spodoptera exigua* Hübner), MBB, potato leafhopper (PLH, *Empoasca fabae* Harris), SBA, SBL, and VBC, was performed to discover novel insect resistance sources. Candidate resistance genes were highlighted for each significant result.

## Materials and methods

### Phenotypic and genotypic data preparation

There were eight cases of insect-related phenotypic data in the GRIN database. Data were categorical records and were transferred to ordinal scales. Phenotypic data of BAW (data contributed by R. L. Nelson), SBL (data contributed by D. Gary, L. Lambert, and R. L. Nelson), and VBC (data contributed by R. L. Nelson) were recorded in five percentage ranges: (i) 0–20% defoliation, (ii) 21–40% defoliation, (iii) 41–60% defoliation, (iv) 61–80% defoliation, and (v) 81–100% defoliation. These five ranges were converted to numbers using 1–5, respectively. Phenotypic data of SBA (Hill et al., 2004; Mensah et al., 2005; Mian et al., 2008; Bhusal et al., 2013; and data contributed by K. Dashiell and L. Hesler) and the defoliation damage caused by unspecified chewing insects (data contributed by L. Hesler) were: (i) resistant, (ii) mostly resistant, and (iii) susceptible. These three categories were converted to 1 to 3, respectively. An original code ranging from 1 to 5, representing little feeding to severe feeding for PLH was used (data contributed by R. L. Nelson). Original values ranging from 1 to 5 (representing damage scales from 1.0 to 1.4, 1.8 to 2.2, 2.6 to 3.4, 3.8 to 4.2, and 4.6 to 5.0, respectively) for MBB were used (Nelson et al., 1987, 1988; Juvik et al., 1989b; Bernard et al., 1998; and data contributed by T. Elden). The record for CEW contained only 27 resistant soybean accessions (Joshi, 1977), and thus the data of CEW was excluded from our study. One agronomic trait, pubescence density, in the GRIN database was also included in our study. The record for pubescence density has six categories from no pubescence to dense pubescence (glabrous, sparse, semi-sparse, normal, semi-dense, and dense), and these categories were converted to 1–6, respectively (Nelson et al., 1987, 1988; Juvik et al., 1989a,b; Coble et al., 1991; Bernard et al., 1998; Hill et al., 2005, 2008; Peregrine et al., 2008; and data contributed by R. L. Nelson). Because the categorical entity challenged the ordinal scale for normality transformation, the raw ordinal phenotypic data were used in this study. Genotypic data were the SNPs derived from SoySNP50K project (Song et al., 2013). Preprocessing of the raw SoySNP50K data was identical to our previous pipeline (Chang et al., 2016a,b,c). Soybean accessions with both phenotypic and genotypic data were included in the association analysis. The number of SNPs with minor allele frequency (MAF) above 0.01 and the sample size (the total number of soybean entries) for each of the seven insects are listed in Table 1.

**Table 1:** **Significant single nucleotide polymorphisms (SNPs) used as fixed covariates in the regular mixed linear model (MLM) for soybean insect resistance and pubescence density based on a genome wide association study**.

Insect/trait^*^	**Sample size^♦^**	**Total SNPs**	**Significant SNP[Table-fn TN3]**	**Chr**.	**Position (Wm82.a1)**	**Position (Wm82.a2)**	***P*-value**	**MAF^#^**	**R^2^ of model without SNP**	**R^2^ of model with SNP**	**FDR-adjusted*P*-value**
BAW	343	37760	–	–	–	–	–	–	–	–	–
Chewing insects	215	38004	–	–	–	–	–	–	–	–	–
MBB	3968	38076	ss715609849	11	27944976	17871050	3.11 × 10^−09^	0.118	0.373	0.379	1.18 × 10^−04^
PLH	771	38200	ss715612746	12	37356120	37312140	6.38 × 10^−21^	0.353	0.308	0.394	1.63 × 10^−16^
Pubescence density	13338	39891	ss715593807	6	18766611	18969749	4.53 × 10^−09^	0.364	0.506	0.507	1.30 × 10^−04^
			ss715604988	9	46139114	49338058	6.62 × 10^−09^	0.433	0.507	0.508	2.64 × 10^−04^
			ss715604998	9	46243163	49446103	1.59 × 10^−08^	0.444	0.508	0.509	6.32 × 10^−04^
			ss715612479	12	34799435	34760529	3.39 × 10^−09^	0.036	0.504	0.506	6.85 × 10^−05^
			ss715612489	12	34877806	34851942	1.81 × 10^−107^	0.423	0.479	0.498	7.20 × 10^−103^
			ss715612493	12	34892779	34866916	9.64 × 10^−24^	0.454	0.501	0.504	3.85 × 10^−19^
			ss715612495	12	34908863	34882999	2.80 × 10^−08^	0.474	0.509	0.511	1.12 × 10^−03^
			ss715612552	12	35246877	35221802	8.93 × 10^−08^	0.089	0.511	0.512	3.41 × 10^−03^
			ss715612488	12	34861072	34835208	5.71 × 10^−10^	0.457	0.512	0.513	2.28 × 10^−05^
			ss715612471	12	34757155	34718187	2.60 × 10^−08^	0.449	0.513	0.514	1.04 × 10^−03^
			ss715612427	12	34477297	34438799	1.08 × 10^−07^	0.200	0.515	0.516	4.31 × 10^−03^
			ss715593807	6	18766611	18969749	4.53 × 10^−09^	0.364	0.506	0.507	1.30 × 10^−04^
			ss715604988	9	46139114	49338058	6.62 × 10^−09^	0.433	0.507	0.508	2.64 × 10^−04^
			ss715604998	9	46243163	49446103	1.59 × 10^−08^	0.444	0.508	0.509	6.32 × 10^−04^
			ss715604810	9	44348623	47548351	8.42 × 10^−08^	0.103	0.514	0.515	2.12 × 10^−03^
			ss715607455	10	44532915	45110885	1.50 × 10^−06^	0.204	0.516	0.517	2.75 × 10^−02^
			ss715634929	19	39828253	40043201	2.13 × 10^−06^	0.397	0.517	0.518	4.62 × 10^−02^
SBA	2075	37952	ss715596142	7	14028462	11259155	2.73 × 10^−76^	0.382	0.455	0.553	1.04 × 10^−71^
SBL	2395	39814	ss715592245	5	4176470	5891372	2.26 × 10^−07^	0.116	0.244	0.253	9.01 × 10^−03^
VBC	445	40000	ss715629577	18	1935991	1936520	1.60 × 10^−06^	0.020	0.165	0.210	4.20 × 10^−02^

* *BAW, beet armyworm, MBB, Mexican bean beetle, PLH, potato leafhopper, SBA, soybean aphid, SBL, soybean looper, and VBC, velvetbean caterpillar*.

♦ *All from Glycine max except for one G. soja entry for BAW, 718 G. soja entries for pubescence, 313 G. soja entries for SBL, and 326 entries for VBC*.

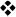
*Significant SNP is defined as the fixed covariate included in the MLM that leads to no more SNPs below FDR 0.05*.

# *Minor allele frequency*.

### Genome-wide association study (GWAS) and linkage disequilibrium (LD) analyses

The R package “GAPIT2” version 2016.03.01 was applied for the association analyses (Lipka et al., 2012; Tang et al., 2016). Kinship was estimated using the VanRaden method (VanRaden, 2008) in a regular mixed linear model (MLM) (Yu et al., 2006). Principal component analysis and Bayesian information criterion (BIC)-based model selection were applied to determine how many principal components (PCs) should be included for additional population structure correction. A false-discovery rate (FDR) using the Benjamini-Hochberg procedure at 0.05 and a Bonferroni-corrected *p*-value at 0.05 were used to determine significance in the multiple association tests. If the first step GWAS identified a significant SNP, the SNP was assigned as a fixed covariate in the MLM for the second step of GWAS. The stepping procedure was stopped when no more significant SNPs were indentified, and SNPs used as fixed covariates were considered as true associated signals. Pairwise LD in the flanking region of the true associated signals were calculated using TASSEL5 with a slide window of 500 bp (Bradbury et al., 2007). Candidate resistance genes were examined within the high LD region centering the significant SNP based on the soybean genome assembly version Glyma.Wm82.a1.v1.1 in SoyBase (http://soybase.org).

## Results

After extracting consensus phenotypic and genotypic data from the GRIN database and the SoySNP50K, there were seven cases for association analyses (BAW, MBB, SBA, SBL, PLH, VBC, and the defoliation damage caused by unspecified chewing insects). For all the cases, a kinship matrix was included in the MLM. Based on the BIC-based model selection, no PC was included in the MLM for any case except for BAW that used three PCs. Among these seven cases, we identified significant signals for five cases, but not for BAW or the defoliation damage caused by unspecified chewing insects (Table 1).

### Mexican bean beetle (MBB)

**Figure 1:**
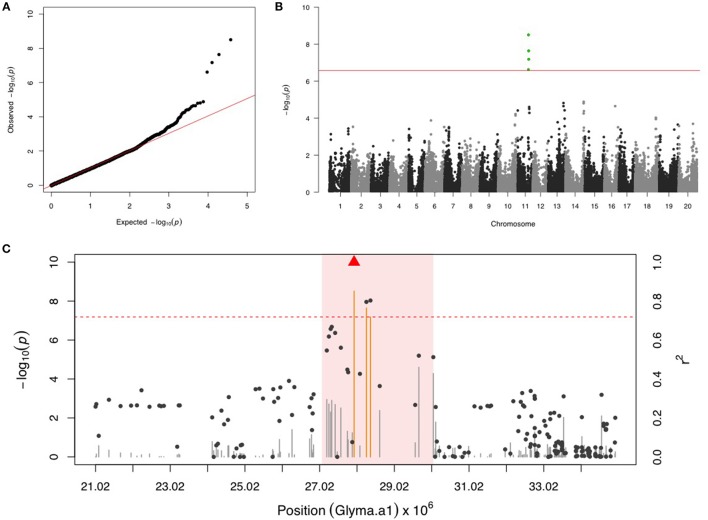
**GWAS for Mexican bean beetle (MBB). (A)** Quantile-quantile plot indicates the fitness of the regular MLM for MBB association analysis. **(B)** Manhattan plot identifies a significant locus on chr 11. Green dots represent the four SNPs with FDR below 0.05. Red horizontal line indicates the Bonferroni threshold. **(C)** Regional LD analysis that surrounds the most significant SNP (ss715609849), which is below the red triangle. The pairwise LD between SNPs (gray dots) to ss715609849 follows the right y axis. The orange and gray color for lines indicates SNPs with FDR below or above 0.05, respectively, following the left y axis. The red horizontal dash line represents the minimal significant level at the cutoff of FDR 0.05. The pink background highlights the high LD region where candidate genes were examined.

There were four SNPs on chr 11 that had a FDR below 0.05 and passed the Bonferonni threshold in the first step GWAS (Figures 1A,B). After including the most significant SNP (ss715609849) as a fixed covariate, the second step GWAS identified no more significant SNPs for MBB (Table 1). LD analysis revealed a relatively high LG condition throughout a 14 MB region centering around ss715609849. Pairwise LD values for most SNPs in this region were between R^2^ values of 0.2 to 0.4, although a region from 27,000,000 to 30,000,000 bp on chr 11 had higher R^2^ values (Figure 1C). There were two genes (Glyma11g29010 and Glyma11g29287) that contained a leucine-rich repeat (LRR) domain within this 3 Mb genomic region.

### Potato leafhopper (PLH)

**Figure 2:**
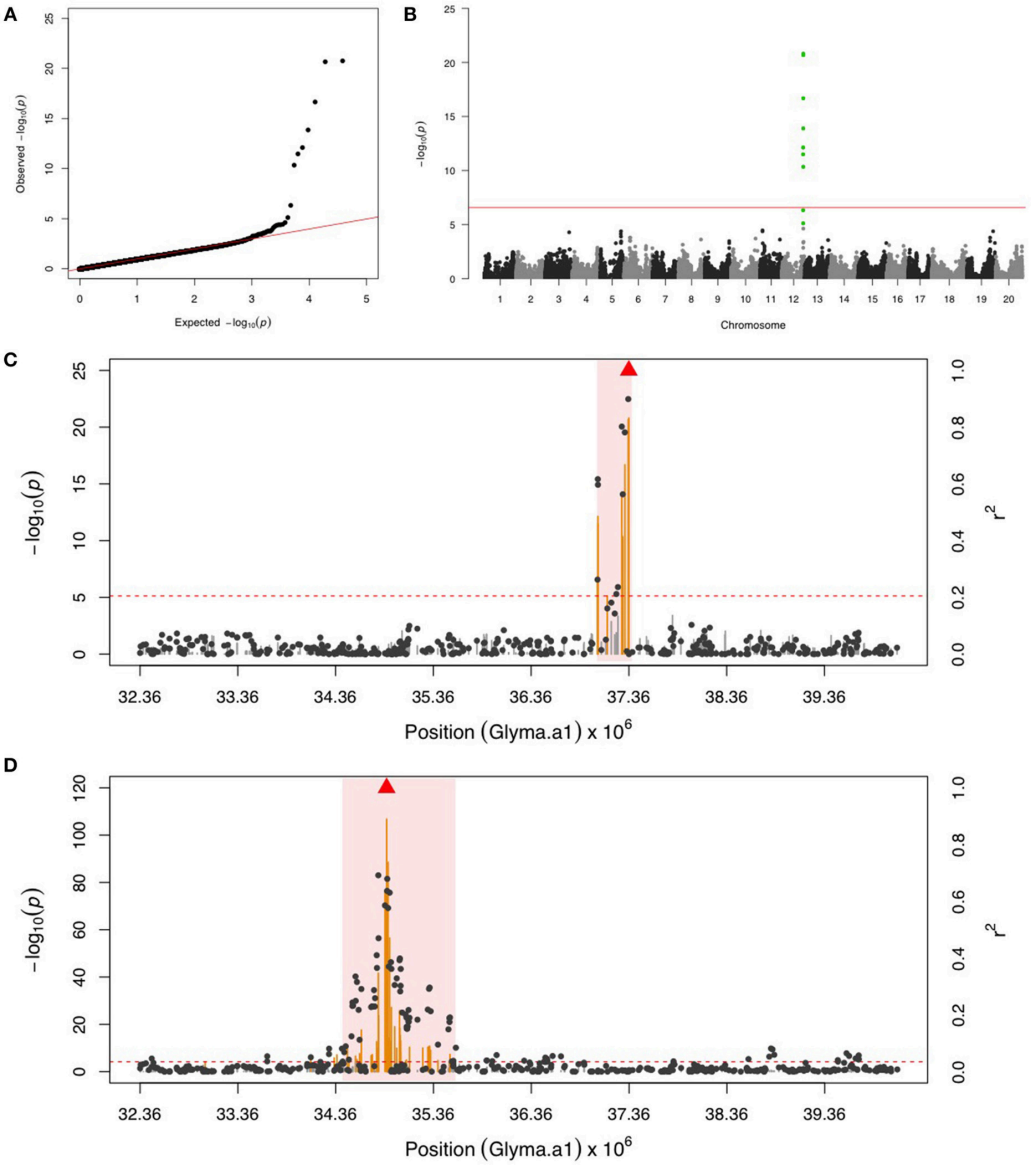
**GWAS for potato leafhopper (PLH) and pubescence density. (A)** Quantile-quantile plot indicates the fitness of the regular MLM for PLH association analysis. **(B)** Manhattan plot identifies a significant locus for PLH resistance on chr 12. Green dots represent the nine SNPs with FDR below 0.05. Red horizontal line indicates the Bonferroni threshold. **(C)** Regional LD analysis for PLH locus that surrounds the most significant SNP (ss715612746), which is below the red triangle. The pairwise LD between SNPs (gray dots) to ss715612746 follows the right y axis. The orange and gray color for lines indicate SNPs with FDR below or above 0.05, respectively, following the left y axis. The red horizontal dash line represents the minimal significant level at the cutoff of FDR 0.05. The pink background highlights the high LD region where candidate genes were examined. **(D)** Regional LD analysis for pubescence density that surrounds the most significant SNP (ss715612489), shown in red triangle. The pairwise LD between SNPs (gray dots) to ss715612489 follows the right y axis. The orange and gray color for lines indicate SNPs with FDR below or above 0.05, respectively, following the left y axis. The red horizontal dashed line represents the minimal significant level at the cutoff of FDR 0.05. The pink background highlights the high LD region where candidate genes were examined. The genomic region for PLH resistance **(C)** and pubescence density **(D)** is exactly the same, but these two loci are not overlapping.

GWAS for PLH identified a locus on chr 12, where nine significant SNPs passed FDR and the Bonferroni threshold (Figures 2A,B). After fixing the most significant SNP (ss715612746) as a covariate, there was no more significant SNPs. This result indicated that the other eight SNPs might be in LD to ss715612746, and indeed the LD analysis found a very narrow interval (around 37,036,017–37,356,120 bp) flanked by these SNPs on chr 12 (Figure 2C), and within this region, there were two LRR domian-containing genes (Glyma12g33930 and Glyma12g34020) and one myeloblastosis (MYB) transcription factor gene (Glyma12g33911). In addition, the location of ss715612746 was within a pubescence density-related QTL 2–7 (qtuH-1) based on the SNP location and QTL 2–7 location from the genome assembly version Glyma.Wm82.a2 (Du and Fu, 2009). In order to understand the genetic architecture of pubescence density in soybean, the phenotype record in the GRIN database was used to run GWAS. We discovered a significant locus on chr 12 (Figures S1A,B); however, the locus for pubescence density ranged from 33 to 36 Mb whereas the locus for PLH was from 37.04 to 37.36 Mb (Figures 2C,D). This indicates that the locus for PLH resistance and the locus for soybean pubescence density do not overlap (Table 1).

### Soybean looper (SBL)

**Figure 3:**
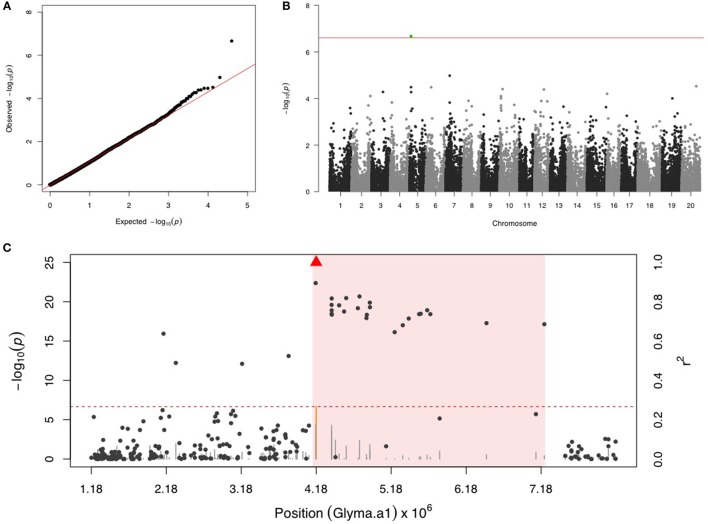
**GWAS for soybean looper (SBL). (A)** Quantile-quantile plot indicates the fitness of the regular MLM for SBL association analysis. **(B)** Manhattan plot identifies a significant locus on chr 5. Green dots represent the one SNP with FDR below 0.05. Red horizontal line indicates the Bonferroni threshold. **(C)** Regional LD analysis that surrounds the most significant SNP (ss715592245), which is below the red triangle. The pairwise LD between SNPs (gray dots) to ss715592245 follows the right y axis. The orange and gray color for lines indicate SNPs with FDR below or above 0.05, respectively, following the left y axis. The red horizontal dashed line represents the minimal significant level at the cutoff of FDR 0.05. The pink background highlights the high LD region where candidate genes were examined.

Only one SNP (ss715592245) was significant on chr 5 for SBL, and there were no more signals after including ss715592245 as a fixed covariate in the second step of GWAS (Figures 3A,B). LD analysis for ss715592245 identified a high LD region starting around the SNP marker toward about a 3 Mb downstream genomic region (Figure 3C). There were four LRR domain-containing genes (Glyma05g05730, Glyma05g06140, Glyma05g06231, and Glyma05g07050) and two MYB transcription factors (Glyma05g04900, Glyma05g06410) located in this high LD region from 4,180,000 to 7,180,000 bp.

### Velvet bean caterpillar (VBC)

**Figure 4:**
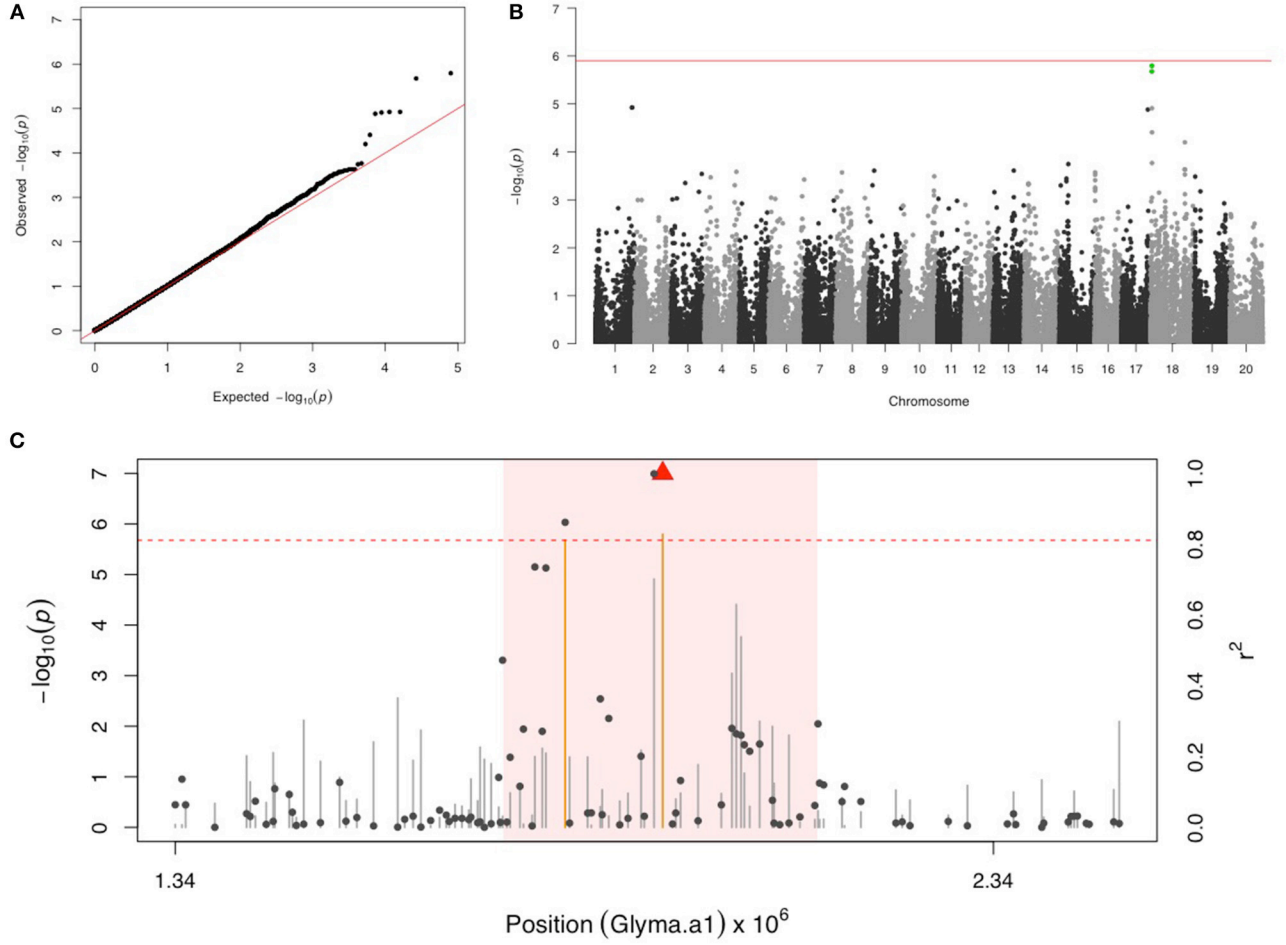
**GWAS for velvetbean caterpillar (VBC). (A)** Quantile-quantile plot indicates the fitness of the regular MLM for VBC association analysis. **(B)** Manhattan plot identifies a significant locus on chr 18. Green dots represent the two SNPs with FDR below 0.05. Red horizontal line indicates the Bonferroni threshold. **(C)** Regional LD analysis that surrounds the most significant SNP (ss715629577), which is below the red triangle. The pairwise LD between SNPs (gray dots) to ss715629577 follows the right y axis. The orange and gray color for lines indicate SNPs with FDR below or above 0.05, respectively, following the left y axis. The red horizontal dashed line represents the minimal significant level at the cutoff of FDR 0.05. The pink background highlights the high LD region where candidate genes were examined.

Two SNPs on chr 18 with a FDR below 0.05 and the significance was close but below the Bonferroni threshold (Figures 4A,B). After fixing the most significant SNP (ss715629577) as a covariate, there were no more significant signals in the second step of GWAS. LD analysis identified a region around 1,740,000–2,126,000 bp that may harbor resistance genes (Figure 4C). There were four LRR domain-containing genes (Glyma18g02850, Glyma18g03040, Glyma18g03053, and Glyma18g03066) located within this 4 Mb LD region.

## Discussion

Soybean resistance mapping using GWAS has been demonstrated on a variety of diseases (Wen et al., 2014; Vuong et al., 2015; Chang et al., 2016c) and the resistance mechanisms have been detailed for many important soybean diseases (Whitham et al., 2016). However, there are just a few GWAS focusing on soybean insect resistance like that of the common cutworm (CCW, *Spodoptera litura* Fabricius) (Wang et al., 2015; Liu et al., 2016). With the availability of GRIN phenotypic data and SoySNP50K genotypic data, the aim of our study was to integrate the information for discovering novel insect resistance sources in the USDA Soybean Germplasm Collection and highlight candidate resistance genes for each insect. In the cases of MBB, PLH, SBL, and VBC, we successfully identified significant SNPs and high LD regions. Because mechanical studies for insect resistance discovered the importance of LRR domain-containing genes (Rossi et al., 1998; Du et al., 2009) and MYB transcription factors (Misra et al., 2010), we reported these two groups of candidate genes within the high LD region. Nonetheless, the possibility that other candidate genes (such as metabolic enzymes or kinases) are involved in insect resistance should not be excluded because insect resistance is complicated and often controlled by multiple mechanisms (Mitchell et al., 2016; Schuman and Baldwin, 2016). Advanced analyses in terms of allelic variation, haplotype diversity, and genomic selection using current SNPs, re-genotyped SNPs with higher marker density, or whole genome re-sequencing data may also provide better understanding of insect resistance in soybean populations (Zhou et al., 2015; Patil et al., 2016). Essentially, molecular cloning and functional analysis for candidate genes are required to confirm their roles in insect resistance.

MBB is a chewing and defoliating insect that damages many legume crops including soybean. MBB is widely distributed in the USA and the southern part of Canada with damage estimates of up to 80% defoliation during the vegetative growth stages of soybean (Nottingham et al., 2016). The soybean cultivar “Davis” was reported to show antixenotic resistance to MBB through coumestrol, an isoflavonoid compound (Burden and Norris, 1992). In addition, two soybean accessions (PI 171451 and PI 229358) were reported to display antibiotic resistance dependent on jasmonic acid regulation (Rufener et al., 1989; Iverson et al., 2001). To the best of our knowledge, there is no genetic mapping study for MBB resistance in soybean or in other host plants. Our study discovered a significant SNP within a 3 Mb high LD genomic region on the chr 11.

The PLH is a major pest in the USA due to its broad host range including alfalfa, common bean, and soybean. Yield losses caused by the PLH were estimated around $66 per hectare for alfalfa (Lamp et al., 1991; Baker et al., 2015) and $2 million for common bean (Gonzales et al., 2002; Brisco et al., 2015). PLH prefers warmer and drier conditions, and a recent study suggested that PLH infestation would be enhanced with increased temperatures due to climate change (Baker et al., 2015). While insecticide application is one way to control PLH, both antibiotic and antixenotic resistance to PLH were found in common bean (Brisco et al., 2015). Some studies on soybean resistance to PLH proposed that antixenotic resistance is governed by pubescence density (Johnson and Hollowell, 1935; Elden and Lambert, 1992), but others suggested additional pubescence characteristics such as orientation (Boerma et al., 1972) or chemicals in the glandular trichomes might be more important than density (Elden and Lambert, 1992; Ranger and Hower, 2001; Peiffer et al., 2009). In the GWAS for PLH, we identified a SNP within the pubescence density QTL 2–7 (qtuH-1), which is close to pubescence density QTL 2–8 (qtuH-2) (Du and Fu, 2009). In addition to this SNP, two genetic markers for soybean CCW resistance were also located within qtuH-1 included Sat_218 found by linkage mapping (Oki et al., 2012) and a SNP (BARC-043061-08513) found by GWAS (Liu et al., 2016). These results indicated qtuH-1 on chr 12 might contribute to CCW and PLH resistance through an antixenotic mechanism that depends on pubescence density. On the other hand, genetic mapping for soybean pubescence density using GWAS in our study provided a better resolution than previous studies using linkage mapping. We showed that the pubescence density locus is distant, about 3 Mb from the PLH locus, and the distance is larger than the average LD size of 0.25 Mb on chr 12 (Vuong et al., 2015). Our results supported the idea that pubescence density may not be the major effect for PLH and another leafhopper (*Empoasca terminalis* Distant) resistance in soybean (Boerma et al., 1972; Nasruddin and Melina, 2014).

SBL has become one of the most problematic pests for soybean production as the insect developed resistance to carbamates, cyclodienes, DDT, organophosphates, permethrin, and pyrethroids (Boethel et al., 1992). While soybean resistance to SBL has been shown to be antixenotic, the resistance depends on the sharp pubescent tip and the *Pb* locus on chr 15 (Hulburt et al., 2004). Another study mapped antixenotic resistance to SBL approximately to QTL-M on chr 7 (Zhu et al., 2008). In addition, antibiotic resistance that relies on chemicals such as afrormosin and phaseol (Caballero and Smith, 1986), growth inhibitors (Smith, 1985; Beach and Todd, 1988), and phytoalexins such as coumestrol and glyceollin were also reported (Liu et al., 1992). Our results discovered a new SNP on chr 5 that may harbor resistance to SBL, and pointed out candidate LRR domain-containing genes.

VBC is another pest that acquired insecticide-resistance (Boethel et al., 1992) and it has been regarded as the most damaging insect for soybean in the southeastern USA (Barbara, 2014). To the best of our knowledge, there is no resistance mapping study for VBC in any crop. Our study identified a 4 Mb LD region on chr 18 with four LRR domain-containing genes. It has been shown that resistance of some soybean cultivars is likely antibiotic and two flavonoids (genistin and rutin) were reported to reduce VBC weights (Piubelli et al., 2005), but other types of candidate genes in the LD region should be considered.

Studies on soybean resistance to the SBA are more abundant than those on all the other insects, and the phenotypic data in the GRIN database contains thousands of accession responses to SBA. Unfortunately, the phenotypic data is unbalanced and only 11 soybean accessions in the GRIN database were classified resistant or mostly resistant. Under the circumstances, GWAS for SBA is problematic because any SNP that is completely shared by these 11 soybean accessions would be considered as a significant signal, and each of these SNPs will co-occur with another SNP. The GWAS result supported this and hundreds of SNPs (Figures S2A,B). When the most significant SNP (ss715596142) was fixed in the second step of GWAS, there were no more significant signals. This observation indicated these SNPs might be confounded with ss715596142 since they were highly correlated to ss715596142 or in LD with ss715596142. We found three LRR domain-containing genes (Glyma07g13440, Glyma07g14810, and Glyma07g14791) and one MYB transcription factor (Glyma07g14480) under a LD region of ss715596142 (from 12,000,000 to 14,800,000 bp) (Figure S2C). However, the location of ss715596142 on chr 7 differs from *Rag1* and the two proposed candidate LRR-containing genes (Glyma07g06890 and Glyma07 g06920) in the *Rag1* locus (Kim et al., 2010a). Instead, ss715596142 is close to *Rag3-1*, which is one of the resistance QTL reported in soybean variety PI 567541B (Zhang et al., 2009). Additional evidence will be needed to support our preliminary analysis for SBA.

## Conclusion

In this study, we report mapping results for the first time for soybean resistance to MBB and VBC located on chr 11 and 18, respectively. While we discovered a novel region for SBL, a locus for PLH that is close to but distinct from the pubescence density locus which also was found. Unfortunately, because of the complexity of insect resistance mechanism and the LD region for the four pests were still to broad, as dozens to hundreds of genes were found that have potential as candidate resistance genes within the LD region. We highlighted two groups of candidate resistance genes that were previously proved for their functions in insect resistance, but advanced studies using additional approaches such as molecular cloning or differential expression analysis may be needed to reduce the candidate resistance gene pool, as was demonstrated for the *Rag2* locus of SBA resistance (Brechenmacher et al., 2015). Our study provided integrated phenotypic and genotypic data and provided a novel insight into soybean insect resistance that might prove useful for insect resistance breeding and reducing insecticide usage.

## Author contributions

HC, Completed data analyses and wrote draft of manuscript. GH, Contributed to writing of manuscript.

## Funding

Research reported in this publication was supported by the USDA Agricultural Research Service.

### Disclosure

Trade and manufacturers' names are necessary to report factually on available data; however, the USDA neither guarantees nor warrants the standard of the product, and the use of the name by USDA implies no approval of the product to the exclusion of others that may also be suitable.

### Conflict of interest statement

The authors declare that the research was conducted in the absence of any commercial or financial relationships that could be construed as a potential conflict of interest.

## Supplementary material

Figure S1 **GWAS for pubescence density. (A)** Quantile-quantile plot indicates the fitness of the regular MLM for pubescence density association analysis. **(B)** Manhattan plot identifies a significant pubescence density locus on chr 12. Red horizontal line indicates the Bonferroni threshold.

Figure S2 **GWAS for soybean aphid (SBA). (A)** Quantile-quantile plot indicates the unfitness of the regular MLM. There are too many SNPs displaying significant signals due to the small pool of resistant soybean varieties. **(B)** Manhattan plot identifies a significant locus on chr 7. Green dots represent the 276 SNPs with FDR below 0.05. Red horizontal line indicates the Bonferroni threshold. **(C)** Regional LD analysis that surrounds the most significant SNP (ss715596142), shown in red triangle. The pairwise LD between SNPs (gray dots) to ss715596142 follows the right y axis. The orange and gray color for lines indicate SNPs with FDR below or above 0.05, respectively, following the left y axis. The red horizontal dashed line represents the minimal significant level at the cutoff of FDR 0.05. The pink background highlights the high LD region where candidate genes were examined.
