# Supplementary figures and images for: Characterization of Insect Resistance Loci in the USDA Soybean Germplasm Collection Using Genome-Wide Association Studies
Source: Front Plant Sci. 2017 May 15;8:670. doi: 10.3389/fpls.2017.00670 (PMC5430066; doi:10.3389/fpls.2017.00670)

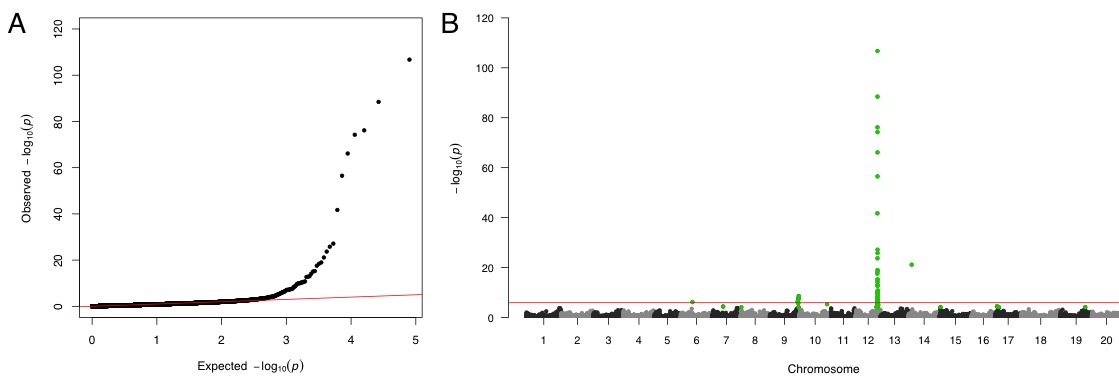

Supplement: Figure S1 — GWAS for pubescence density. (A) Quantile-quantile plot indicates the fitness of the regular MLM for pubescence density association analysis. (B) Manhattan plot identifies a significant pubescence density locus on chr 12. Red horizontal line indicates the Bonferroni threshold. [file Image1.JPEG]

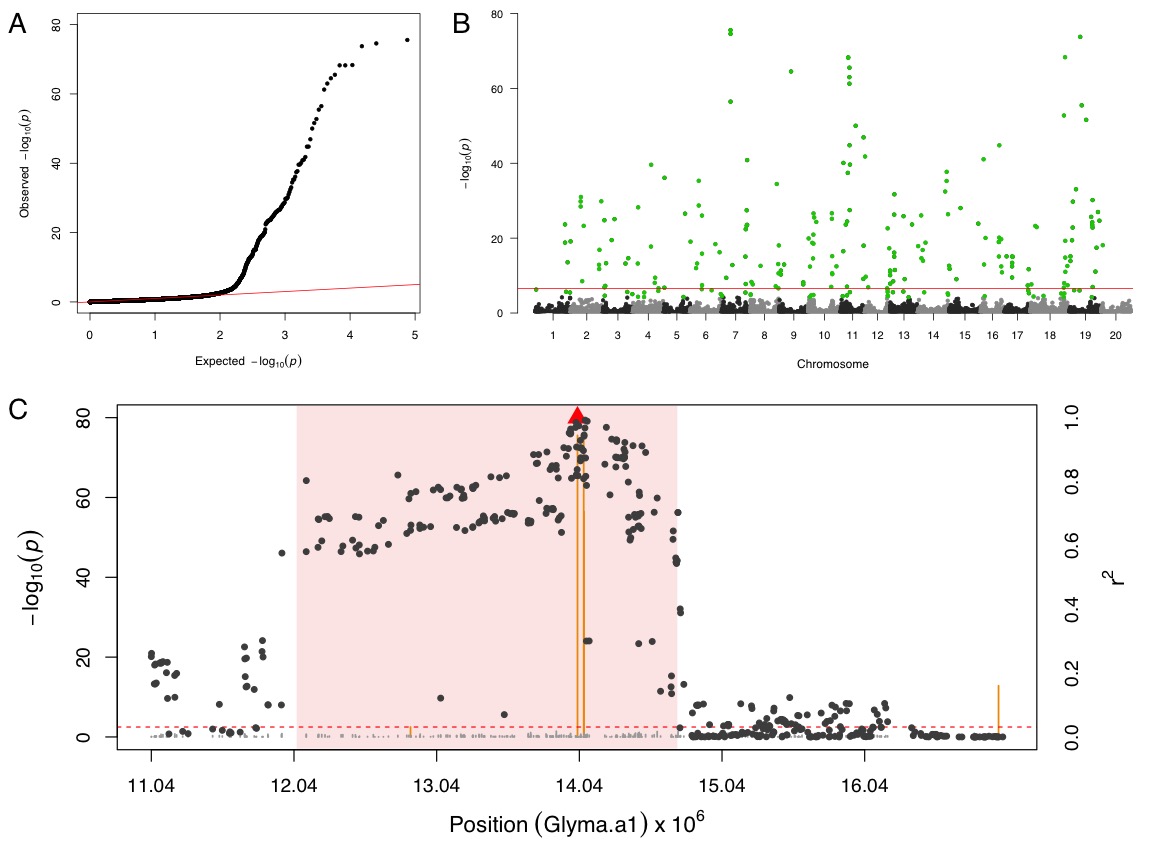

Supplement: Figure S2 — GWAS for soybean aphid (SBA). (A) Quantile-quantile plot indicates the unfitness of the regular MLM. There are too many SNPs displaying significant signals due to the small pool of resistant soybean varieties. (B) Manhattan plot identifies a significant locus on chr 7. Green dots represent the 276 SNPs with FDR below 0.05. Red horizontal line indicates the Bonferroni threshold. (C) Regional LD analysis that surrounds the most significant SNP (ss715596142), shown in red triangle. The pairwise LD between SNPs (gray dots) to ss715596142 follows the right y axis. The orange and gray color for lines indicate SNPs with FDR below or above 0.05, respectively, following the left y axis. The red horizontal dashed line represents the minimal significant level at the cutoff of FDR 0.05. The pink background highlights the high LD region where candidate genes were examined. [file Image2.JPEG]
